# *De Novo* Assembly of *Candida sojae* and *Candida boidinii* Genomes, Unexplored Xylose-Consuming Yeasts with Potential for Renewable Biochemical Production

**DOI:** 10.1128/genomeA.01551-15

**Published:** 2016-01-14

**Authors:** Guilherme Borelli, Juliana José, Paulo José Pereira Lima Teixeira, Leandro Vieira dos Santos, Gonçalo Amarante Guimarães Pereira

**Affiliations:** a Departamento de Genética, Evolução e Bioagentes, Instituto de Biologia, Universidade Estadual de Campinas, Campinas, SP, Brazil; b Dangl Lab, Department of Biology, University of North Carolina, Chapel Hill, North Carolina, USA; c GranBio/Biocelere Agroindustrial, Campinas, SP, Brazil

## Abstract

*Candida boidinii* and *Candida sojae* yeasts were isolated from energy cane bagasse and plague-insects. Both have fast xylose uptake rate and produce great amounts of xylitol, which are interesting features for food and 2G ethanol industries. Because they lack published genomes, we have sequenced and assembled them, offering new possibilities for gene prospection.

## GENOME ANNOUNCEMENT

*Candida boidinii* and *Candida sojae* are both xylose-consuming yeasts from the *Saccharomycetales* order of *Ascomycota*. *C. boidinii* was also found as a methylotrophic yeast (1), while *C. sojae* lacks further studies of its metabolic pathways. *C. boidinii* was first identified from a wash of tree barks (2), and recently, we have isolated it from sugarcane bagasse. *C. sojae* was first isolated from a liquid fraction of water-soluble substances of defatted soybean flakes, in Japan (3), and we have isolated it from *Diatraea saccharalis* (*Lepidoptera*), a plague-insect in an energy-cane cultivar.

From the yeast strains we have isolated, these *Candida* isolates were shown as the best strains in xylose consumption (*C. boidinii* and *C. sojae*) and in xylitol production (*C. sojae*; unpublished results), a sugar-alcohol interesting for food industry as a sweetener (4). In addition, genes related to xylose uptake and in xylitol production are target of interest in the 2G ethanol industry, since xylose is a sugar present in abundance in lignocellulosic substrates and xylitol is an intermediate of ethanol production from xylose (5). There was no exploration of biotechnological potential of these yeasts and both had no published genome. Thus, we have extracted their genomes and sequenced, assembled, and analyzed them, searching for genes from xylose metabolism (5).

Samples were sent to the University of North Carolina at the High-Throughput Sequencing Facility (HTSF) for genome sequencing using an Illumina HiSeq2000, which produced a library with insert sizes around 400 nucleotides (nt) and near to 116 million paired reads for *C. boidinii* and 109 million for *C. sojae*, with 100 nt each. Considering their reduced genome sizes, we conducted the assembly using the SPAdes version 3.5 pipeline (6).

Initial read coverage (900×) was reduced by randomly sorting reads into one-third. The default SPAdes pipeline worked very well for *C. boidinii*, but a first assembly round for *C. sojae* suggested that we were dealing with a polypoid genome. We proceeded with the *C. sojae* assembly using the dipSPAdes module, which resulted in a high-quality assembly for a consensus haploid genome. The *C. boidinii* final assembly comprised approximately 19 Mb organized into 428 scaffolds, with an *N*_50_ of 49 sequences and a GC content of 30.7%. The *C. sojae* final assembly comprised approximately 12 Mb organized into 511 scaffolds, with an *N*_50_ of 65 sequences, and a GC content of 32.4%. Final read coverage for the assembled genome was 144× for *C. boidinii* and 48× for *C. sojae*.

Gene predictions were performed using Augustus version 3.2.1 (7) with the previously available training set for *Candida tropicalis*. For *C. boidinii*, 5,978 genes with an average sequence length of 548 amino acids were identified, while for *C. sojae* these numbers were 52,31 genes with an average length of 466 amino acids.

### Nucleotide sequence accession numbers.

The *Candida sojae* whole-genome shotgun project has been deposited at DDBJ/EMBL/GenBank under the accession number LMTL00000000. The version described in this paper is the first version, LMTL01000000.

The *Candida boidinii* whole-genome shotgun project has been deposited at DDBJ/EMBL/GenBank under the accession number LMZO00000000. The version described in this paper is the first version, LMZO01000000.
